# Effects of different analgesic methods used for vaginal delivery on mothers and fetuses

**DOI:** 10.3906/sag-1911-61

**Published:** 2020-06-23

**Authors:** Gülçin BABAOĞLU, Banu KILIÇASLAN, Aysun ANKAY YILBAŞ, Bilge ÇELEBİOĞLU

**Affiliations:** 1 Department of Anesthesiology and Reanimation, Faculty of Medicine, Hacettepe University, Ankara Turkey

**Keywords:** Labor pain, meperidine, neuraxial analgesia

## Abstract

**Background/aim:**

Knowledge regarding pain relief during labor remains insufficient. We aimed to determine and compare the effectiveness and safety of epidural analgesia, combined spinal–epidural analgesia, and parenteral meperidine on both mothers and fetuses.

**Materials and methods:**

This study was designed as an observational case-control study. We collected prospective data from patients whose labor pain management was conducted with meperidine in addition to retrospective cohort data of neuraxial methods; 138 patients were enrolled. Epidural analgesia group consisted of 68 patients, whereas combined spinal-epidural (CSE) analgesia group and meperidine group consisted of 50 and 20 patients, respectively. We compared the delivery patterns, labor durations, pain levels, side effects, maternal satisfaction levels, and neonatal outcomes of the various pain management methods.

**Results:**

Patient demographics, duration of first, second, and third labor stages, and instrumental delivery rates were comparable among groups (P > 0.05). Cesarean section tended to be less frequent in the CSE group. In the meperidine group, visual analog scale (VAS) values and sedation were significantly higher (P < 0.001) and maternal satisfaction lower (P < 0.001). Hypotension tended to be more frequent in the meperidine group. APGAR scores at the 1st and 5th min were similar among the groups and between meperidine subgroups defined by three different administration times (<1 h, 1‒4 h, ≥4 h; P > 0.05).

**Conclusion:**

Neuraxial methods had no effect on instrumental delivery rates. CSE represented a near significant risk reduction in cesarean section. Our results demonstrated that regional analgesia methods were reasonably safe for both mother and fetus, and regional analgesia methods resulted in greater maternal satisfaction and pain control compared to meperidine.

## 1. Introduction

Labor pain is one of the most painful experiences for most women. It alters respiratory, cardiovascular, neuroendocrine, and limbic systems and may lead to adverse outcomes as a result. In addition, it triggers psychodynamic behaviors resulting in stress and anxiety [1], which might cause metabolic acidosis and decreased uteroplacental blood flow [2]. Achieving sufficient pain relief is a significant factor for successful labor and maternal satisfaction. 

Numerous pharmacological and nonpharmacological methods are currently being used to reduce labor pain [3]. The choice of analgesic method depends on the expectations of the mother, the joint decision of anesthesiology and reanimation and the obstetrics clinics, and the progress of labor [4]. Neuraxial methods provide almost perfect pain control when the mother is fully awake and cooperative [5]. The side effects of these methods on the maternal cardiovascular-pulmonary system and fetal physiology are considered minimal [6]. However, there is conflicting information regarding the issue. There are studies reporting that neuroaxial analgesia significantly increases the cesarean rate [7,8], whereas some studies reported the contrary [9,10]. Furthermore, controversies remain regarding the risk of instrumental delivery. Instrumental deliveries are associated with various long-term disadvantages, including an increased likelihood of fecal incontinence, sexual dysfunction, and hospital stay extensions [11–13]. Recent trials have reported the lowered risk of instrumental delivery with modern low-dose epidural regimens; however, these regimens do not completely mitigate these adverse outcomes. 

Systemic analgesics may be used for pain relief in labor. The commonest opioid used for labor is meperidine [14], administered by intramuscular (im) injection. Meperidine remains the standard of care for labor pain in some clinics; it can also be used at the request of the patient or in cases when neuraxial analgesia is not feasible. However, current knowledge remains uncertain. Some studies have reported an increase in the frequency of the neonatal depression when delivery occurred after 2‒3 h after meperidine administration [15] while some studies have not [16,17]. Furthermore, there is a gap of knowledge in terms of maternal satisfaction with meperidine treatment [18]. 

In 2018, a Cochrane review assessed the effectiveness and safety of all types of epidural analgesia on mothers and infants compared to nonepidural methods. They reported the superior efficacy of epidural analgesia in reducing pain and improving maternal satisfaction. In addition, they reported that studies regarding this topic provided low-quality evidence, limited by inconsistency and imprecision and underlined the need for more robust research to evaluate possible maternal and fetal effects, in particular, side effects and maternal satisfaction[18].

In view of the controversies and knowledge gap existing in the literature, we aimed to determine the effectiveness and safety of epidural analgesia, combined spinal–epidural analgesia, and parenteral meperidine on both the mother and fetus.

## 2. Materials and methods 

### 2.1. Patient selection, inclusion, and exclusion criteria

This study was designed as a case-control study. Informed consent was obtained from patients. This study was approved by the ethical committee of Hacettepe University (Date: 24/06/2015- Number: 16969557-721 NO: GO 15/376-15). After the approval of the local ethic committee, the files of patients who had regional analgesia for labor pain were analyzed retrospectively. Among 167 cases, 49 cases were excluded due to missing data (*n *= 35), gestation week lower than 37 weeks or higher than 41 weeks (*n* = 4 and *n* = 1, respectively), catheter problems (extraction of the catheter due to complication, *n* = 3; catheter dislocation *n* = 1), and spinal anesthesia (*n* = 5). Eventually, the retrospective arm comprised 118 patients, including 68 patients who were administered with epidurals and 50 patients with CSE analgesia. In addition to the retrospective cohort data, we collected the prospective data of patients whose labor pain management was done with meperidine. Twenty patients who were unwilling to have neuraxial analgesia and given instead meperidine according to the standard protocol were included in the study. Patients who were in gestational periods less than 37 or higher than 41 gestational weeks were excluded in addition to those with fetal presentation and multiple pregnancies.

### 2.2. Data collection

We have applied a standard labor pain protocol in our clinic since 2012. In accordance with this protocol, we meticulously recorded data from all patients, including the following: 

· demographic data (age, height, weight, number of births, gestational age, and presence of comorbid diseases);

· intervention information (local anesthetic agent, opioid solution content, application technique, and administration method [infusion/bolus/infusion and bolus]); 

· complications, presence, and duration of motor-sensorial block; 

· cervical dilatation and contraction rate at the moment of catheter insertion, durations of the Stages 1, 2, and 3;

· amount of drug used during infusion, bolus amount, other drug use, the number of bolus need; 

· hemodynamic data (pulse, systolic and diastolic arterial pressure, and fetal heart rate after 5, 10, 15, 30, 45, 60, 90 min, and every following half hour);

· delivery patterns (vaginal/cesarean section), usage of vacuum or forceps, indication for cesarean section, if there is any;

· general pain evaluations (visual analogue scale [VAS] scores prior to application, 15th min, first and second stage, at the time of episiotomy, and fetal expulsion);

· maternal satisfaction levels (based on a 5-point Likert scale);

· newborn outcomes (weight, first and 5th min Apgar scores, intensive care unit [ICU] admission, and reasons for admission to ICU).

### 2.3. Standardized application methods

For the CSE technique, 15‒25 µg of fentanyl with 2.5 mg bupivacaine was used for spinal analgesia. Continuous infusion was started half an hour after the spinal application. In the pure epidural technique, bolus 0.125%‒0.25% bupivacaine (10 mL) was given after the test. In both methods, after the block placement, patient-controlled analgesia (PCA) with baseline infusion followed. The baseline rate was set to 8‒12 mL/h, the bolus to 5 mL, the lock duration to 10 min, the hourly limit to 30 min with a solution of 1%‒2% µg/fentanyl and 0.0625% bupivacaine. 

We considered birth a natural process. If patients demanded pain control, we offered neuraxial analgesia as the standard treatment for labor pain. Patients who did not want neuraxial analgesia and those administered meperidine for labor pain were enrolled as the prospective observational group; 25 mg intravenous (IV) and 50 mg intramuscular (IM) meperidine were administered to these patients, respectively. Aforementioned variables, with the exception of the intervention and regional block information, were also recorded for those patients administered meperidine.

### 2.4. Statistical methods

The data were analyzed with SPSS 21.0 for Windows. We examined the suitability of the numerical variables to normal distribution using visual (histogram and probability graphs) and analytical methods (the Kolmogorov–Smirnov/Shapiro–Wilk tests). We made comparisons of numerical data between groups using analysis of variance (ANOVA) or Kruskal–Wallis (where appropriate). A 5% type I error level was used to infer statistical significance. Further pairwise comparisons were analyzed with post hoc Tukey test or Mann–Whitney U-tests using Bonferroni correction and setting the statistical significance at 1.67% type I error level (where appropriate). p1 represents hypothesis between the epidural and CSE groups; p2, between CSE and meperidine group; and p3, between the epidural and meperidine groups. We compared nominal data with Chi-square or Fisher’s test. We presented descriptive analyses as mean ± standard deviation (s.d.) or median (minimum and maximum) values according to distribution. Exceptional cases were specified on the tables. The minimum number of participants required was determined using power analysis. In order to detect a minimum clinically significant difference of one-unit change on the satisfaction scale with a statistical power at the 80% level and 5% type-1 error, we selected a minimum of 20 patients for meperidine treatment and at least 50 participants for each of the other two groups.

## 3. Results

Table 1 details the demographic and clinical characteristics of the patients. Epidural treatment included 68 patients, CSE treatment included 50 patients, and the meperidine group included 20 patients. The mean age of the patients was 27.3 ± 4.9 years, and the mean gestational age was 39 ± 1.0. Age, body mass index (BMI), gestational age, nulliparity, ASA classification, and cervical dilatation at the time of catheter insertion were comparable between the groups. No significant difference between drugs’ doses (infusion/bolus/infusion+bolus) in epidural and CSE groups (P > 0.05) were detected.

**Table 1:** Patient characteristics stratified by analgesic methods.

	Total (n = 138)	Epidural (n = 68)	CSE (n = 50)	Meperidine (n = 20)	P-value
Age (y)x	27.3 (4.9)	27.2 (4.4)	27.3 (5.5)	27.5 (4.8)	0.97
BMI (kg/m2) x	28.4 (3.7)	28.2 (4.0)	29.2 (3.2)	27.5 (3.6)	0.23
Gestational age (wk) x	39 (1.0)	39 (1.0)	392 (1.2)	39 (1.1)	0.74
Nulliparity (%)	58	57	56	65	0.78
ASA I/II/III	122/12/4	62/4/2	45/5/0	15/3/2	0.11
Cervical dilatation at catheter insertion (cm) *	3 (0–6)	3 (0–6)	3 (2–5)	3 (1–6)	0.14
Need for cesarean section	38 (28%)	22 (32%)	8 (16%)	8 (40%)	0.06

xData represented as mean (SD), *Data represented as median (min–max) BMI = Body mass index, CSE = Combined spinal epidural, ASA=American Society of Anesthesiologists.

Table 2 outlines labor and delivery characteristics. One hundred patients (72.5%) delivered their infant via normal vaginal delivery, and 38 (27.5%) ended up with cesarean sections. The duration of the vaginal delivery stages was similar between groups (P > 0.05). Instrumental delivery was used only for one patient in the epidural group (P = 0.55). There was a trend towards to decrease of risk in the cesarean delivery rate in the CSE group (p1 = 0.04, p2 = 0.06, and p3 = 0.52).

**Table 2:** Labor and delivery characteristics of patients.

	Epidural	CSE	Meperidine	P-value
Normal spontaneous vaginal delivery (n=100)	n = 46	n = 42	n = 12	
Duration of first stage, h	11.5 (2.5-102)	9 (1.5-24.5)	6.75(3-22.5)	0.22
Duration of second stage, min	9(2–20)	10(5–30)	10(2–15)	0.57
Duration of third stage, min	5(2–15)	5(2–20)	8.5(2–11)	0.12
Vacuum-forceps usage	1 (2.2%)	-	-	0.55
Cesarean delivery (n = 38)*	n = 22	n = 8	n = 8	
Deceleration (n = 21)	11(50%)	6(75%)	4(50%)	0.55
Arrest of labor (n = 13)	8(36%)	2(25%)	3(38%)	0.91
Uterine tetany (n = 1)	1(5%)	-	-	
Patient’s own will (n = 1)	-	-	1(12%)	

*Missing data, the indication of caesarean section could not be reached from files of two patients. CSE; Combined spinal epidural

Among patients who were applied neuraxial methods, those who gave birth through vaginal delivery were divided into two groups according to the width of cervical dilatation at the time of catheter insertion: <4 cm (*n* = 45) and ≥4 cm (*n* = 42). There was no statistically significant difference among the groups regarding the proportion of the nulliparity (P = 0.76). The duration of the first stage was 12 h (min–max, 2.5‒28.5) in patients with cervical dilatation measuring <4 cm at the time of catheter insertion, 7.25 h (min–max, 1.5‒36) in patients with cervical dilatation measuring ≥4 cm. The duration of the first stage was significantly longer in the group with cervical dilatation measuring <4cm (P = 0.012). The duration of the second stage was similar (10 min, 2‒30; 7 min, 5‒20, respectively; P = 0.123).

Cesarean section was performed due to deceleration in the nonstress test (NST) (*n* = 21), arrest of labor (*n* = 13), patients own will (*n *= 1), an unknown indication (*n* = 2), and uterine tetany (*n* = 1). The cesarean section rates due to deceleration or arrest of labor were comparable between groups (P > 0.05). Maternal satisfaction and VAS values at the 15th min, first stage, second stage, time of episiotomy, and time of pushing were differed between groups (P < 0.001). Pairwise comparisons demonstrated that VAS values in the meperidine group were significantly higher, whereas maternal satisfaction was lower (P < 0.001) (Figure). 

**Figure:**
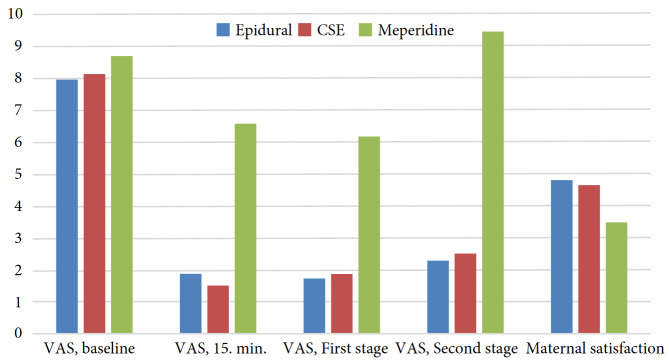
VAS values of the groups at different time points.

Table 3 details the motor block and side effects of the various methods. Motor block and pruritus was comparable between groups (P = 0.28 and P = 0.81, respectively). Sedation was more frequent in the meperidine group, whereas hypotension was more common in the CSE and epidural groups. Frequency of nausea, vomiting, shivering, need for ephedrine use, and back pain were similar among groups. The 1st- and 5th-min Apgar scores were similar between the epidural, CSE, and meperidine groups (P = 0.97 and P = 0.23, respectively). Apgar scores in the 1st and 5th min were not affected by the elapsed time between meperidine administration and birth (0‒1 h, 1‒4 h, and >4 h). Six infants all of whom were delivered with epidural analgesia (P = 0.047) were admitted to the neonatal intensive care unit due to respiratory distress (*n* = 2), small gestational age (*n* = 2), and unknown indication (*n* = 2).

**Table 3:** Comparison of motor block and adverse events between groups.

	Epidural n = 68	CSE n = 50	Meperidine n = 20	P-value
Motor block	17(25%)	8(16%)	-	-
Pruritus	1(1.5%)	1(2%)	-	0.81
Sedation	-	-	6(30%)	<0.001
Nausea	4(5.9%)	2(4%)	3(15%)	0.23
Vomiting	2(2.9%)	3(6%)	1(5%)	0.79
Hypotension	15(22.1%)	10(20%)	-	0.07
Ephedrine use	13 (19%)	7 (14%)	-	0.18
Shivering	2(2.9%)	-	-	0.35
Back pain	2(2.9%)	2(4%)	-	0.67

CSE; Combined Spinal Epidural

## 4. Discussion

The pain of labor ranks consistently among the most severe types of pain that a woman will experience during life. From the expert’s point of view, the gold standard for pain relief in labor is neuraxial analgesic techniques. However, meperidine is still used as a standard of care in many clinics and there are conflicting results in the literature regarding the efficacy and safety of these methods. Considering these conflicts and gap of knowledge within the literature, reporting the results of the effects of epidural, combined spinal epidural, and parenteral meperidine on mothers and fetuses remain crucial. The present study has addressed these controversies. 

First, we discovered similar instrumental delivery rates among groups. Cesarean delivery rates tended to be low in the CSE group. Despite the high-quality analgesia and the high maternal satisfaction, reservations remain regarding the rate of instrumental delivery and cesarean section in patients who were administered neuraxial analgesia [7–9]. In a metaanalysis involving 2400 patients, epidural analgesia increased the duration of the first and second stages of labor, unlike the risk of cesarean section [10]. Another metaanalysis suggested similar instrumental delivery rates between combined spinal–epidural and epidural analgesia [7]. Both instrumental delivery and cesarean section rates of neuraxial analgesia were reported to be lower with diluted local anesthetics [19]. Although they could not report a definite statement, a recent Cochrane review indicated that an increased rate of instrumental delivery was more likely to be related with less modern epidural techniques [18]. In accordance with this result, we could not identify any increase in instrumental delivery rates with our technique, which may be considered modern, considering the low concentration mixture of a local anesthetic and opioid (0.0625% bupivacaine and fentanyl 2 μg/mL) that we used. Besides the techniques used, the variable rates of instrumental and cesarean delivery could also be explained by the experience of the center and the history of the patient population.

Second, we found that regional analgesic methods did not affect the duration of the various labor stages in the whole study population. However, subgroup analysis revealed that the first stage of delivery was lengthened for patients whose cervical dilatation was less than 4 cm at the time of catheter insertion. In accordance with our findings, studies have indicated that regional anesthesia that were initiated when cervical dilatation was less than 4 cm prolonged labor [20,21]. On the contrary, a randomized controlled study showed that regional anesthesia had no effect on labor durations, instrumental delivery, or cesarean section rates, even when neuraxial intervention was applied for under 2 cm of cervical dilatation [22]. 

Third, we found the decrease in the VAS values were significantly higher in patients whose pain management was done with epidural or CSE analgesia compared to the meperidine group. These results are in agreement with previous studies [23–25]. Indeed, our results are noteworthy. We selected patients who expressed an explicit desire not to have an epidural analgesia; in that case, the prospect of access and knowledge to better care could not bias results. Although patient satisfaction was significantly lower in the meperidine group, it was better than our expectations. This may be due to the sedation and drowsiness caused by meperidine. In a metaanalysis from 2010 [15], it was shown that sedation and somnolence were more frequent in patients who were on meperidine. There was no difference among the groups in terms of the 1st- and 5th-min Apgar scores of the newborns of patients, which is also consistent with the literature [7,24,26–28].

Fourth, we demonstrated that the administration time of meperidine did not affect the fetal outcomes. It has been shown that the peak plateau level of the fetal concentration of meperidine appeared 1‒5 h after administration. Although reports on the issue have been conducted, the clinical significance of different administration times have not been proven yet [15]. In our study, considering meperidine pharmacokinetics [29], patients who completed vaginal delivery were divided according to elapsed time between the birth and the meperidine administration (<1 h, 1‒4 h, and ≥4 h). First- and fifth-minute Apgar scores were similar in these groups.

The neonatal ICU admission rates were higher in the epidural group. This result was somewhat unexpected since the neonatal intensive care admission rates are known to be similar among the different analgesia groups [27]. Selection bias might explain this since three of the newborns were already scheduled for neonatal intensive care follow-up before the initiation of labor. However, as per the reported limitations of the recent Cochrane review [18], it is important to report any kind of side effects that may be associated with neuraxial analgesia. 

Ours is one of the very few studies that have analyzed the effects of all three techniques on the entire range of outcomes related to the mother and fetus. However, the retrospective nature of the neuraxial groups remained a major limitation. 

Our study sought to respond to the contradictions in the literature and reported the efficacy and side effects of each method meticulously. We demonstrated that regional analgesia methods were reasonably safe for both mother and fetus and that regional analgesia methods resulted in greater maternal satisfaction and pain control compared to meperidine. Extensive patient monitoring and accurately chosen methods with appropriate dosing of local anesthetics could minimize the side effects of neuraxial methods. 

## Conflict of interest

The authors have no related conflict of interest to disclose.
